# Enhancement of microbial fuel cell performance by introducing a nano-composite cathode catalyst

**DOI:** 10.1016/j.electacta.2018.01.118

**Published:** 2018-03-01

**Authors:** Mounika Kodali, Sergio Herrera, Sadia Kabir, Alexey Serov, Carlo Santoro, Ioannis Ieropoulos, Plamen Atanassov

**Affiliations:** a Department of Chemical and Biological Engineering, Center Micro-Engineered Materials (CMEM), MSC01 1120 University of New Mexico Albuquerque, New Mexico, 87131, USA; b Bristol BioEnergy Centre, Bristol Robotics Laboratory, T-Block, UWE, Coldharbour Lane, Bristol, BS16 1QY, UK; c Biological, Biomedical and Analytical Sciences, UWE, Coldharbour Lane, Bristol, BS16 1QY, UK

**Keywords:** Oxygen reduction reaction (ORR), Microbial fuel cell, PGM-Free, Graphene nanosheets, Iron catalyst

## Abstract

Iron aminoantipyrine (Fe-AAPyr), graphene nanosheets (GNSs) derived catalysts and their physical mixture Fe-AAPyr-GNS were synthesized and investigated as cathode catalysts for oxygen reduction reaction (ORR) with the activated carbon (AC) as a baseline. Fe-AAPyr catalyst was prepared by Sacrificial Support Method (SSM) with silica as a template and aminoantipyrine (AAPyr) as the organic precursor. 3D-GNS was prepared using modified Hummers method technique. The Oxygen Reduction Reaction (ORR) activity of these catalysts at different loadings was investigated by using rotating ring disk (RRDE) electrode setup in the neutral electrolyte. The performance of the catalysts integrated into air-breathing cathode was also investigated. The co-presence of GNS (2 mg cm^−2^) and Fe-AAPyr (2 mg cm^−2^) catalyst within the air-breathing cathode resulted in the higher power generation recorded in MFC of 235 ± 1 μW cm^−2^. Fe-AAPyr catalyst itself showed high performance (217 ± 1 μW cm^−2^), higher compared to GNS (150 ± 5 μW cm^−2^) while AC generated power of roughly 104 μW cm^−2^.

## Highlights

• Fe-AAPyr and GNS were synthesized and characterized.
• Rotating ring disk electrode (RRDE) setup was performed in the neutral electrolyte.
• Fe-AAPyr had higher half wave potential and lower H_2_O_2_ production.
• The combination of Fe-AAPyr and GNS led to a power generation of 235 ± 1 μWcm^−2^.
• Both Fe-AAPyr and GNS outperformed compared to activated carbon control.

## 1 Introduction

Energy plays a vital role in the everyday life. With the increasing energy demands, the effort has focused on developing low cost and efficient, energy harvesting technologies utilizing renewable energy sources. Microbial fuel cell (MFC) has been extensively studied in the past decade due to the co-generative characteristics of removing organic contaminants and producing valuable electricity output [[1], [2], [3], [4]]. In fact, MFCs belong to the bioelectrochemical systems (BES) technology [[1], [2], [3], [4]], a larger family that comprises of electrochemical technologies having a biotic anode in which the oxidation reaction occurs [[1], [2], [3], [4]]. MFCs work by converting the chemical energy stored in the organics into electrical energy by the aid of electroactive microorganisms that act as the biological catalysts at the anode. At the anode electrode, the organic material, which is the anodic fuel, is oxidized and therefore degraded generating the electrons that are released to the anode electrode [5]. These electrons get carried through an external load to the cathode where an oxidant (i.e. oxygen in air breathing MFCs) is reduced, and the overall redox reaction is then completed [[1], [2], [3], [4], [5]]. Despite several oxidant options investigated in MFCs [6], oxygen is the preferred solution due to its natural availability in the atmosphere at no additional cost and its high electrochemical potential [6].

The overall MFC performance is affected by various factors such as operating conditions (e.g. temperature [7,8], pH [9,10], solution conductivity [11,12], etc.), electrode materials [[13], [14], [15]] and their structure [16,17], cell design [18,19], microbial inoculation [[20], [21], [22]] etc. Despite the low electricity production, several demonstrations of harvesting towards applicability have been showed in existing literature [[23], [24], [25], [26], [27]]. Few examples of application comprehend robots prototypes [28,29], sensors [[30], [31], [32], [33], [34], [35], [36]], watch [37] and LEDs lights [38].

Although MFCs have been studied in detail in the past decade, one of the major limitations that are still impeding its large-scale applications can be attributed to the poor cathode kinetics operating in neutral media [[39], [40], [41], [42], [43], [44], [45]]. In this solution conditions, in fact, H^+^ and OH^−^, both necessary reagents for the ORR, are present in a concentration of 10^−7^ M which is the lowest possible in the available pH range. In general, ORR can occur by a direct 2e^−^ or 4e^−^ transfer mechanism, or an indirect 2x2e^−^ transfer mechanism and this depends from the catalysts material utilized during the reduction reaction [[42], [43], [44], [45]]. The nature of the electrolyte solution, especially the pH, employed during the ORR also drives the reaction pathways towards different intermediate and final products [[42], [43], [44], [45]]. Mainly, in acidic conditions, O_2_ can be reduced to H_2_O (direct 4e^−^), to H_2_O_2_ (direct 2e^−^) or H_2_O towards a 2x2e^−^ transfer mechanism with H_2_O_2_ as intermediate. In alkaline electrolyte, O_2_ can be reduced to OH^−^ (direct 4e^−^), HO_2_^−^ and OH^−^ (direct 2e^−^) or OH^−^ towards a 2x2e^−^ transfer mechanism with HO_2_^−^ and OH^−^ as intermediate. In the case of intermediate generation, the latter can be chemically or electrochemically transformed into H_2_O or OH^−^ in case of acidic or alkaline media electrolyte respectively [[42], [43], [44], [45]].

Due to the low kinetics, additional catalysts are used to accelerate the overall reaction. The catalysts might be of the biotic or abiotic type. The first one considers enzymatic [46] and microbial [41] catalysts. Several enzymes have been exploited as catalysts for ORR in neutral media with the superb performance, but both high cost and low durability make those materials unsuitable for MFCs applications [47]. Microbial catalysts (aerobic [48] and anaerobic [[49], [50], [51]]) are also studied, and it was found that they enhance the ORR of graphite-based cathodes. Still, the mechanism is quite complex and not completely understood. Two main families of materials instead belong to the category of abiotic catalysts. Those families are named: i) platinum-group metal (PGM) [52,53]; ii) platinum-group metal-free (PGM-free) [[54], [55], [56], [57]]. The main difference is the presence or absence of platinum group metals such as ruthenium (Ru), rhodium (Rh), palladium (Pd), osmium (Os), iridium (Ir), and platinum (Pt). PGM-free catalysts can also be subdivided into two categories comprehending carbonaceous-based materials [[54], [55], [56], [57]] or earth abundant transition metals-based materials [[54], [55], [56], [57]].

More predominantly, PGM catalysts were used in the initial stages of developing the cathode materials for MFCs [52,57]. The reason for this can be attributed to the utilization of already advanced electrodes from a more mature fuel cell technology such as acidic (e.g., PEMFC, DMFC, etc.) or alkaline (e.g., AFC, etc.) fuel cells [54,57,58]. Due to their high cost, their utilization should be minimized or avoided in low performing MFCs. Besides this, the possibilities of PGM catalysts of getting poisoned in MFC are very high since the catalysts are directly exposed to wastewater, activated sludge (AS) and other organics/pollutants which contains large amounts of cations and anions that interact directly with the catalyst active centers, reducing dramatically the electrocatalytic activity [[59], [60], [61]].

Those limitations resulted in the development of platinum group metal free (PGM-free) catalysts as cathode materials for MFCs applications. Compared to PGM catalysts, PGM-free catalysts are more resistant towards poisoning [[59], [60], [61]] and much lower cost to be used in MFC [62,63]. Carbonaceous-based materials such as activated carbon (AC) [[64], [65], [66], [67], [68], [69]], carbon nanotubes (CNT) [70], carbon nanofibers (CNF) [71], 2D or 3D-graphene nanosheets [72], etc. [73] were also used as cathode materials due to their low-cost, high surface area, relatively high conductivity and durability in “harsh” and polluted environments. The performance of carbonaceous-based materials is very limited compared to PGM-free catalysts containing earth abundant transition metals such as Fe [[74], [75], [76], [77], [78], [79], [80], [81], [82], [83], [84]], Mn [[85], [86], [87]], Co [[87], [88], [89]] and Ni [87,90,91]. Therefore, carbonaceous materials are often used as support for the catalyst rather than be the catalyst itself. PGM-free containing metals are generally: i) oxides; ii) macro-cycles (e.g. phthalocyanine or porphyrins) in which a metal center is incorporated; iii) product of pyrolysis of a metal salt and an organic precursor. Pyrolyzed PGM-free catalysts are generally the most used for MFCs [84]. It was shown previously that Fe-based catalysts were most active among Co-, Mn- and Ni-based materials in both rotating ring disk electrode (RRDE) [87] and in working MFCs [82].

The objective of this work is to examine the performance of activated carbon (AC), graphene nanosheet (GNS), and iron-aminoantipyrine (Fe-AAPyr) catalyst materials separately and integrated as an alternative cathode catalyst material to improve MFCs performance. The electrocatalytic activity of AC, GNS and Fe-AAPyr in neutral media using RRDE as well as incorporated into air-breathing cathodes was investigated. Polarization and power curves of operating MFCs having air-breathing cathodes with the catalysts integrated were also studied and discussed. The effect of the addition of GNS and Fe-AAPyr separately and simultaneously was shown and discussed.

## 2 Experimental

### 2.1 Catalyst synthesis

#### 2.1.1 Fe-AAPyr synthesis

Fe-AAPyr catalyst was synthesized using Sacrificial Support Method (SSM) as reported previously [59,62,63]. Iron nitrate and Aminoantipyrine were ball-milled with Silica template till achieving a fine powder. The obtained mixture was then pyrolyzed at a temperature of 950 °C for 30 min under Ultrapure Nitrogen gas at a flow rate of 100 mL min^−1^. The temperature was ramped up to 950 °C using a rate of 25 °C min. After pyrolysis, the mixture was cooled down to room temperature under atmospheric conditions in the furnace. Silicate was removed from the catalyst (etching) by the utilization of hydrofluoric acid (HF) of 20 wt%, and the catalyst was thoroughly washed with DI water to attain the neutral pH. Thus, remained silica free catalyst was dried at a temperature of 85 °C facilitating the water evaporation.

#### 2.1.2 Fabrication of three dimensional GNS

To synthesize the graphene nanosheets, first its precursor, graphene oxide (GO_x_) was synthesized using the Modified Hummers method [92]. The GO_x_ solution was then exfoliated using high energy ultrasonic solution and impregnated with monodispersed amorphous fumed silica (Cab-O-Sil^®^ L90, surface area ≈90 m^2^ g^−1^) was dispersed into the solution that was further ultrasonicated for 1 h. The GO-silica mixture was then dried overnight, followed by ball milling and thermal pyrolysis in 7% H_2_ (flow rate = 100 mL min^−1^) at a controlled temperature of 800 °C for the duration of 1 h. The obtained GNS-Silica material was ball milled again at 400 rpm for 15 min. The GNS-silica reduced powder (graphene nanosheets containing silica nanoparticles) was subjected to HF etching in order to remove the silica particles used as template, hence giving it a porous three-dimensional morphology The powder was leached in 40 wt% HF overnight and then washed until neutral pH was achieved, followed by air drying (T = 85 °C), and additional pyrolysis in inert UHP nitrogen atmosphere at a temperature of T = 850 °C for 2 h. The fabrication and comprehensive characterization of these three-dimensional graphene nanosheets was established in previous publications [[93], [94], [95]].

### 2.2 Rotating ring disk electrode experiments (RRDE)

Catalytic ORR performance of AC, GNS, Fe-AAPyr, and Fe-AAPyr-GNS was studied by RRDE. The RRDE was composed by a disk of glassy carbon with an area of 0.2475 cm^2^ that was surrounded by a platinum ring with an area of 0.1866 cm^2^. Ring and disk were connected to a different channel of a bi-potentiostat (Pine Technology). Precisely 5 mg of the catalysts were weighted into 2 mL individual Eppendorf tubes (Table 1). In case of Fe-AAPyr-GNS catalyst mixture, 2.5 mg of each catalyst was added to the tube (Table 1). Into these tubes, 150 μL of 0.5 wt% Nafion solution and 850 μL of 4:1-DI water and Isopropanol were added and sonicated for about 15 min in the ultrasonic bath to disperse the catalyst inks homogeneously. Before the tests, the catalyst inks were re-sonicated again for about 3 min in pulse mode with 30 s' intervals in the middle by using micro-tip ultrasonic probe. Three different loadings of all the catalysts (0.2, 0.4, and 0.6 mg cm^−2^) were tested in 0.1 M neutral potassium phosphate buffer (K-PB) solution of 7.5 pH with AC as the control. Only one test was performed for each type of catalyst presented in Table 1. Before carrying out the RRDE experiment, the buffer solution was purged thoroughly with pure oxygen gas for at least 20 min to saturate the electrolyte with dissolved oxygen (DO). The catalyst ink was drop cast on to the glassy carbon disk (WE) and dried completely before attaching it to the shaft. Linear Sweep Voltammograms (LSVs) was performed by using the general three-electrode cell assembly with the glassy carbon electrode (with the dry catalyst on top) as the working electrode, graphite rod as the counter electrode and Ag/AgCl 3 M KCl as the reference electrode. LSVs were run between +0.5 V and −0.7 V at a scan rate of 5 mVs^−1^ at a rotation rate of 1600 rpm. While running the LSVs, the disk current (I_disk_) and the ring current (I_ring_) were recorded to find the electrochemical parameters of interest such as onset potential, half-wave potential and the limiting current of all the catalysts. The amount of peroxide generated (eq. (1)) and the number of electrons transferred (eq. (2)) during the reduction reaction were also calculated from the I_disk_ and I_ring_ using the formulas below:

(1) $${\text{\%H}}_{2}{\text{O}}_{2}=\frac{200\;\text{x}\;\frac{\;I\;ring}{N}}{I\;disk+\;\frac{I\;ring}{N}}$$

(2) $$\text{n}=\frac{4\;\text{x}\;I\;disk}{I\;disk+\;\frac{I\;ring}{N}}$$

where N = 0.43, which represents the platinum ring collection efficiency that was given by the instrument company.

**Table 1:** Loadings of AC, GNS, Fe-AAPyr and Fe-AAPyr-GNS catalysts on working electrode (WE) for RRDE experiments.

Catalysts	mg in the ink	Loadings (mg cm^−2^)		
**AC**	5	0.2	0.4	0.6
**GNS**	5	0.2	0.4	0.6
**Fe-AAPyr**	5	0.2	0.4	0.6
**Fe-AAPyr-GNS**	2.5 + 2.5	0.2	0.4	0.6

### 2.3 Electrodes preparation

Anode electrodes utilized in this work were two carbon brushes (3 cm in diameter and 3 cm in height) that were already in use for more than one year resulting in fully-grown and operating electroactive biofilm on the electrodes. Cathodes were fabricated in the form of a circular pellet by using a metallic pellet dye under the pressure of 3 mT applied for 5 min using a hydraulic press (Carver, USA). A ratio of 7:1:2 of activated carbon (AC), carbon black (CB), and polytetrafluoroethylene (PTFE) were taken and blended to form a uniform mixture using a blender. Then the AC/CB/PTFE mixture was blended with 20 mg of the catalysts (AC, GNS, Fe-AAPyr, and Fe-AAPyr-GNS mixture) and made into air-breathing cathodes respectively. The compositions of the cathodes investigated are also presented in Table 2.

**Table 2:** Electrode configuration with loading of AC/CB/PTFE, GNS and Fe-AAPyr.

Catalysts	Loadings (mg cm^−2^)			
	AC/CB/PTFE	GNS	Fe-AAPyr	Total
AC-0	380	–	–	380
AC-2	420			420
GNS-2	400	20	–	420
Fe-AAPyr-2	400	–	20	420
Fe-AAPyr-2-GNS-2	380	20	20	420

### 2.4 Cathodes polarization curves and microbial fuel cell polarization curves

The prepared cathodes were screwed to a lateral hole of the glassy MFC and filled with 0.1 M of potassium phosphate buffer solution (K-PB) of 7.5 pH to carry out the linear sweep voltammetry (LSV) measurements. This type of single chamber membraneless microbial fuel cells was previously presented and fully described [96,97]. The cathode was left in direct contact with the electrolyte overnight. LSV was carried out at a scan rate of 0.2 mV s^−1^ from open circuit potential (OCP) to −0.4 V (vs. Ag/AgCl) with the cathode as working electrode, titanium wire (>2 m in length) as the counter electrode and Ag/AgCl (3 M KCl) as the reference electrode. Triplicates of different air-breathing cathodes were run for each catalyst investigated.

The solution was switched with 50:50 - K-PB and activated sludge (AS) along with 3 mL of sodium acetate (NaOAc) solution (stock of 100 g L^−1^) as bacterial feed. The AS was taken from the Southside Wastewater Reclamation Plant, Albuquerque, NM, USA. As the solution was switched, the anodes were moved into the “new” MFCs, and the overall system was left in open circuit voltage (OCV) for at least 3 h before running the overall polarization curve.

The polarization curves were run from OCV to 0 V at a scan rate of 0.2 mVs^−1^. Triplicates of different MFCs were run for each catalyst investigated. While doing the polarization curves, individual potentials of cathode and anode were measured separately using another potentiostat channel. The cathode surface area exposed to the solution was 2.85 cm^2^. All the calculations were referred to the geometric cathode area exposed to the electrolyte.

## 3 Results and discussion

### 3.1 Surface morphology

**Fig. 1:**
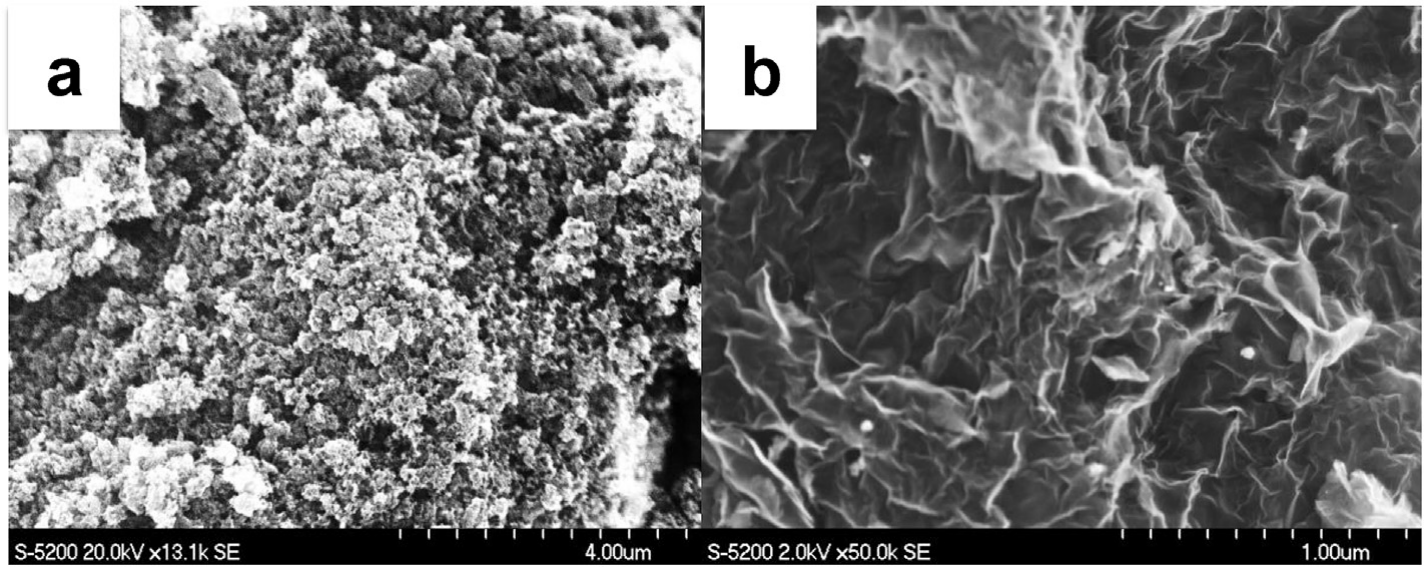
SEM images of Fe-AAPyr (a) and 3D-GNS (b).

Fig. 1 shows the SEM images of a) Fe-AApyr and b) three dimensional GNS materials of the materials utilized in this study. The porous morphology was generated via thermal pyrolysis and etching of the silica template, and also verified using BET and BJH N_2_-isotherms. The Fe-AApyr catalyst was shown to have a BET surface area of 650 m^2^ g^−1^, whereas the three-dimensional GNS had a BET surface area of 300 m^2^ g^−1^.

### 3.2 RRDE data

**Fig. 2:**
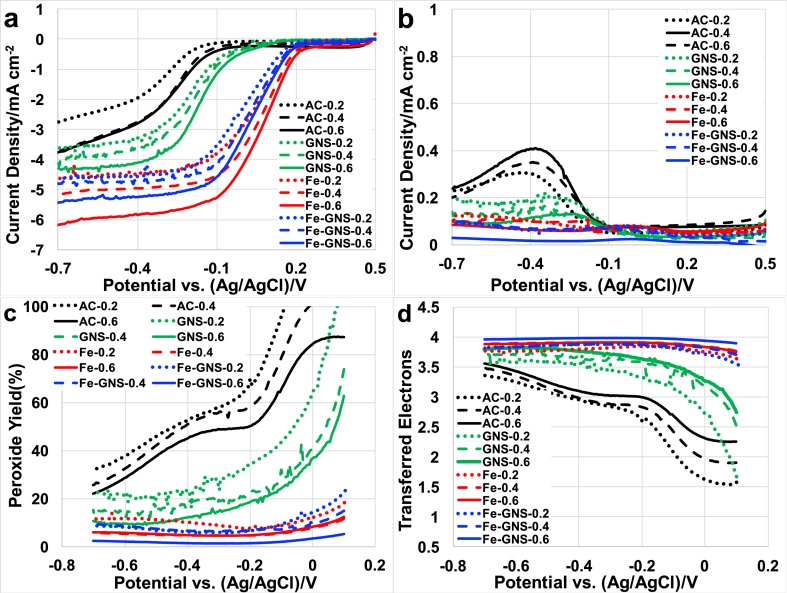
Disk current (a), ring current (b), peroxide yield (c) and electron transfer number (d) at rotating speed of 1600 rpm for AC, GNS, Fe-AAPyr and Fe-GNS (Fe-AAPyr + GNS) catalysts at loadings of 0.2, 0.4 and 0.6 mg cm^−2^.

The disk current for the AC, GNS, Fe-AAPyr and Fe-AAPyr-GNS catalysts at different loadings is shown in Fig. 2 a. The onset potential varies for different catalysts and is approximately ≈ +0.23 V (vs. Ag/AgCl) for Fe-AAPyr and Fe-GNS catalysts, while for AC it is roughly ≈ −0.075 V (vs. Ag/AgCl) and for GNS is ≈ +0.05 V (vs. Ag/AgCl). As expected, the onset potential remained similar for all catalysts independently of their loading. This agrees with the previously reported data [62,72,82,84]. The half-wave potential is highest for Fe-AAPyr of about +0.075 to +0.01 V (vs. Ag/AgCl) and the lowest is for AC of about −0.25 to −0.32 V (vs. Ag/AgCl). For GNS and Fe-GNS, the half-wave potential ranges between −0.17 and −0.21 V (vs. Ag/AgCl) and +0.025 to +0.06 V (vs. Ag/AgCl) respectively. The limiting current also follows the same pattern followed by the half-wave potential. From the disk current, Fe-AAPyr-GNS performance was quite comparable with the Fe-AAPyr even though the quantity of Fe-AAPyr within the Fe-AAPyr-GNS catalyst is half the quantity compared to the Fe-AAPyr catalyst by itself. By increasing the catalysts loading the limiting current was increased for all the catalysts, this might be due to entrapping and quick conversion of generated peroxide in the catalyst layer itself before it gets released in the electrolyte. But irrespective of loading the onset potential remained the same while the half-wave potential increased slightly as shown in Table 3.

**Table 3:** Half-wave potentials of all the catalysts at different loadings.

Catalyst Loading (mg cm^−2^)	Fe-AAPyr-GNS (V vs Ag/AgCl)	Fe-AAPyr (V vs Ag/AgCl)	GNS (V vs Ag/AgCl)	AC (V vs Ag/AgCl)
0.2	0.025	0.075	−0.204	−0.32
0.4	0.04	0.09	−0.195	−0.275
0.6	0.055	0.1	−0.175	−0.275

The ring current was also measured, and it increased gradually in case of AC and GNS as the potential goes down from 0 to −0.7 V (vs. Ag/AgCl) indicating the presence/formation of H_2_O_2_ in the electrolyte as ORR intermediate shown in Fig. 2 b. Ring current density (I_ring_) measured had a minor values for Fe-AAPyr-GNS and Fe-AAPyr comparatively among all the catalysts indicating a complete reduction of oxygen to the final product during the ORR and lower production of peroxide as intermediate.

Peroxide yield calculated according to eq. (1) was also shown in Fig. 2 c. Low peroxide yield quantified in roughly ≈10–20% at +0.1 V (vs Ag/AgCl) and ≈5–10% at −0.7 V (vs. Ag/AgCl) was measured for Fe-AAPyr-GNS and Fe-AAPyr (Fig. 2c). Instead, GNS produced much higher peroxide yield of about 60–100% at +0.1 V that decreased to 10–22% at lower potential investigated (−0.7 V vs. Ag/AgCl). Even higher peroxide was measured in the case of AC that was ≈85–100% at +0.1 V (vs. Ag/AgCl) and ≈20–35% at −0.7 V (vs. Ag/AgCl). For both AC and GNS, the peroxide production increased with the decrease in the potential, and this can be interpreted by the fact that initially (at high potential) peroxide is produced and then is consumed once the potential moves towards lower values. Interestingly, peroxide yield was also decreased with the increasing loading for all the catalysts tested in this study. With the utilization of higher loading, the catalyst layer thickness on the glassy carbon electrode increased resulting in less amount peroxide reaching the ring of the electrode. This means that all the catalysts do not have a direct 4e-transfer mechanism but all of them produce intermediate products that are then consumed by the thicker layer before reaching the ring.

The number of electrons transferred during the reaction was shown in Fig. 2 d. Fe-AAPyr-GNS is having the highest number of e-transferred of about 3.75–4 in number. For Fe-AAPyr, the number of e^−^ transferred was 3.7–3.9, 3.5–3.7 for GNS, and 3.35–3.5 for AC with different loadings. Fe-AAPyr-GNS and Fe-AAPyr had always high e^−^ transferred while for AC and GNS, the e-transfer number increased at lower operating potentials. As for every catalyst investigated, a not negligible peroxide production was measured, it can be speculated that a direct 4 e^−^ transfer mechanism was not present within the catalysts investigated. Fe-AAPyr-GNS and Fe-AAPyr follow a 2 × 2 e^−^ mechanism while AC and GNS follow more probably a direct 2 e^−^ transfer mechanism mainly due to the high peroxide produced at higher potential.

### 3.3 LSVs in electrolyte

**Fig. 3:**
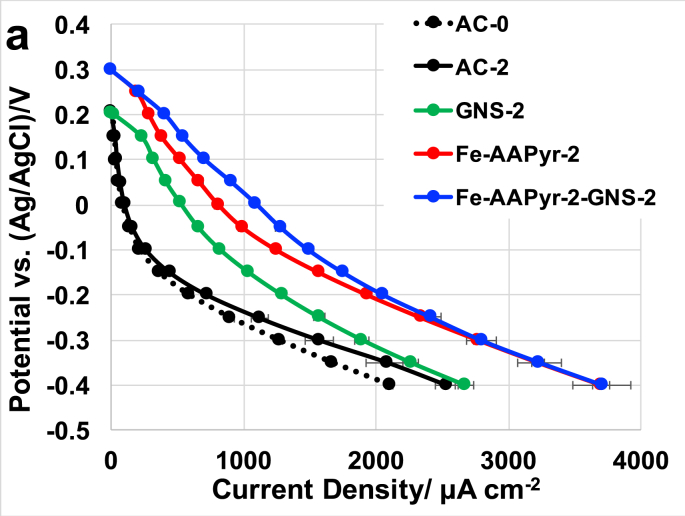
LSVs of AC, GNS, Fe-AAPyr and Fe-GNS catalysts incorporated into air breathing cathode.

Fig. 3 shows the LSV studies done on the air-breathing cathodes with different catalysts combination to understand their electrocatalytic activity under clean conditions using 0.1 M K-PB as the electrolyte solution. After leaving the cells filled with K-PB solution overnight, these experiments were conducted; this initial conditioning resulted in obtaining a stable open circuit potential (OCP) for all the cathodes investigated. The OCPs of Fe-AAPyr-2 and Fe-AAPyr-2-GNS-2 catalysts were around 0.32 V (vs Ag/AgCl) while AC-0, AC-2 and GNS-2 was around 0.2–0.25 V (vs Ag/AgCl) (Fig. 3). This indicates that the presence of atomically dispersed iron moieties within the catalyst shifted up the cathode OCP. At low current densities, AC underwent much higher activation losses compared to GNS and Fe-AAPyr based catalysts. GNS-based cathodes always had higher current output compared to AC despite at lower potentials their current outputs became comparable. The addition of Fe-AAPyr produced more current compared to the Fe-free cathodes (Fig. 3). The addition of GNS within the Fe-AAPyr (Fe-AAPyr-2-GNS-2) helped to enhance the catalytic activity of the cathode within over 2000 μA cm^−2^ of current generated performing then like Fe-AAPyr-2 air-breathing cathode. None of the catalyst containing cathodes underwent concentration losses during the experiment at higher current densities indicating that ohmic losses were predominant at those currents level. Fe-AAPyr-2-GNS-2 and Fe-AAPyr-2 cathode reached a maximum current density of 3700 μAcm^−2^ (at −0.4 V vs Ag/AgCl), in parallel, AC (AC-0 and AC-2) and GNS-2 have attained a maximum current density of 2000–2500 μA cm^−2^ and 2700 μA cm^−2^ (at −0.4 V vs Ag/AgCl).

### 3.4 Polarization and power curves in operating microbial fuel cells

Overall polarization curves were performed on the MFCs after the cells attained the stable OCV. The OCVs of the MFCs were measured before running the overall polarization curves with values of about 0.73–0.75 V for Fe-AAPyr-2 and Fe-AAPyr-2-GNS-2. Instead, the OCV for GNS-2, AC-0, and AC-2 was lower and quantified in around 0.65 V and 0.67 V (Table 4).

**Table 4:** Open circuit voltage, maximum power density and short circuit current density for the MFCs during overall polarization curves.

Catalyst	Open circuit	Max. power	Short circuit current
	voltage (V)	density (μW cm^−2^)	density (μA cm^−2^)
AC-0	0.64 ± 0.01	103 ± 4	992 ± 43
AC-2	0.67 ± 0.01	105 ± 1	932 ± 34
GNS-2	0.67 ± 0.01	150 ± 5	1172 ± 23
Fe-AAPyr-2	0.74 ± 0.02	218 ± 5	1355 ± 10
Fe-AAPyr-2-GNS-2	0.74 ± 0.01	235 ± 1	1500 ± 4

**Fig. 4:**
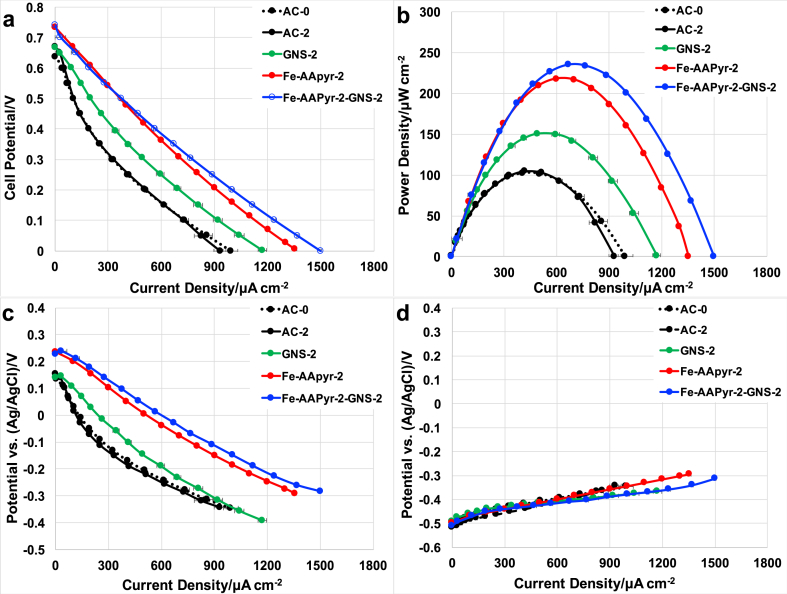
Overall polarization curves (a), power curves (b), anode polarization (c) and cathode polarization curves (d) of the MFCs having AC-0, AC-2, GNS-2, Fe-AAPyr-2 and Fe-AAPyr-2-GNS-2 catalysts.

Fig. 4 a shows the overall polarization curves of the MFCs with the different catalysts incorporated into an air-breathing cathode. Fe-AAPyr-2-GNS-2 obtained the maximum short-circuit current density of about 1500 ± 3.5 μA cm^−2^ while Fe-AAPyr-2 and GNS-2 achieved a lower short-circuit current density of about 1355 ± 10 μA cm^−2^ and 1172 ± 23 μA cm^−2^ respectively. AC-based cathodes MFCs had instead the lower short circuit current densities of about 932 ± 34, 992 ± 43 μA cm^−2^ for 2 (AC-2) and 0 (AC-0) mg cm^−2^ additional loadings. The power densities (Fig. 4b) were obtained from the polarization curves by multiplying the voltage and current density (P=V × I). Fe-AAPyr-2-GNS-2 achieved the maximum power density of this investigation of about 235 ± 1 μW cm^−2^ while Fe-AAPyr-2 achieved a slightly lower power density of about 218 ± 5 μW cm^−2^. GNS-2 based cathode MFCs achieved maximum power densities of about 150 ± 5 μW cm^−2^. AC-0 and AC-2 had similar power density peak of 103 ± 4 μW cm^−2^ and 105 ± 1 μW cm^−2^ respectively. To elucidate the behavior of the single electrodes during the polarization curves, cathode and anode performance were recorded separately (Fig. 4c and 4 d). Anode polarization curves showed very similar electrochemical characteristics for all the electrodes utilized (Fig. 4c). This anode trend confirms therefore that the differences detected during the polarization curves are imputable to the diverse cathodes used during the experimentation. Cathode polarization curves for Fe-AAPyr-2-GNS-2 were slightly better than Fe-AAPyr-2. As they started from the same OCP, Fe-AAPyr-2-GNS-2 cathodic curves had slightly lower slopes indicating the lower ohmic resistance of the material that can be attributed to the presence of the highly conductive GNS. GNS-2 cathode MFC had higher cathode performance compared to AC that had similar behavior despite different loading. Cathode and anode polarization curves, as well as the overall polarization curves, did not suffer from any transportation or diffusion loss even at higher current densities investigated indicating the major losses occurring in the cells are corresponding to ohmic losses.

## 4 Outlook

In this work, different cathode catalysts were investigated in air-breathing cathode MFC. The catalysts used such as AC, GNS, Fe-AAPyr, and GNS mixed with Fe-AAPyr were initially screened using RRDE technique for identifying the kinetics parameters of the catalyst towards ORR. RRDE allows identifying the catalyst having the best performance within its working regime of saturated oxygen in the liquid electrolyte. Fe-AAPyr showed highest performance followed by GNS and AC. While a clear 2e^−^ transfer mechanism was identified for GNS and AC, supported by their high peroxide production, a more complicated 2 × 2 electron transfer mechanism can be considered for Fe-AAPyr. This speculation was supported by the small but detectable hydrogen peroxide produced by Fe-AAPyr. An increase of loading leads to a lower peroxide production indicating that the intermediate formed by the reaction is disproportionated within the thicker catalyst layer. A mixture of AC, CB and PTFE was used as the base for every cathode investigated. At that base, AC, GNS, Fe-AAPyr, and GNS mixed with Fe-AAPyr were added as catalyst, and the electrochemical performance were studied in clean media and in operating MFC.

Fe-AAPyr-2-GNS-2 measured the highest power reached in this investigation that was 235 ± 1 μW cm^−2^. Fe-AAPyr-2 had a comparable but slightly lower power density of about 218 ± 5 μW cm^−2^. The addition of GNS within the cathode increased the overall performance by 8%. GNS-2, AC-0, and AC-2 had a lower power density. Particularly, the addition of extra AC did not affect the performance output while the addition of GNS boosted up the performance by 50%. The addition of Fe-AAPyr and Fe-AAPyr and GNS simultaneously lead to advancement in performance that was 2-fold compared to the AC. Once again, it can be concluded that the addition of catalyst based on iron on or graphene nanosheets can increase the performance significantly compared to simple AC.

In literature, PGM-free catalysts incorporated into cathodes and tested in membrane less MFCs using a buffer solution as overall or part of the electrolyte (maximum considered of 0.1 M PBS buffer concentration) showed performance between 150 and 280 μW cm^−2^ [[74], [75], [76], [77], [78], [79], [80], [81], [82], [83], [84], [85], [86], [87], [88], [89], [90], [91], [92]]. This big variation among the results in literature is mainly due to the different operating conditions utilized during the experimentations such as temperature, electrolyte adopted, cell design, anode/cathode ratio, altitude on sea level, etc [11,82]. Other parameters such as catalyst loadings, for example, have been showed to affect the power output [62]. At last, it was previously shown that Fe-based catalysts performed better compared to another PGM-free catalyst in which Co-, Mn- and Ni-was adopted as the metal center [82,87]. Comparing these performance with previously presented catalysts tested in the exactly same operating conditions and with the same loading applied, the higher performance here presented in this current work (235 ± 1 μW cm^−2^) represent one of the highest reported in neutral media. Fe-Mn-AAPyr showed a peak of power curves of 222 ± 7 μW cm^−2^ [98], Fe-Nicarbazin [84] instead had a maximum power of 214 ± 5 μW cm^−2^. Slightly lower power generation that was still above 200 μW cm^−2^ was reached by Fe-Ricobendazole and Fe-Niclosamide [60]. In parallel, higher performance (243 ± 7 μW cm^−2^) were achieved when iron (II) phthalocyanine was deposited into black pearl carbon black and incorporated into an air-breathing cathode [75]. The latter catalyst was not done using sacrificial support method. This work underlined the importance of designing new cathode catalyst materials that can enhance the overall performance of the MFC system significantly.

## 5 Conclusions

The electrocatalytic activity towards oxygen reduction reaction of activated carbon (AC), graphene nanosheets (GNS) and iron-aminoantipyrine (Fe-AAPyr) were tested using rotating ring disk electrode (RRDE) technique. Fe-AAPyr had higher electrochemical output compared to GNS that was also superior to AC. Higher peroxide was detected for AC and GNS indicating a probable 2e^−^ transfer mechanism. Fe-AAPyr had lower but not negligible H_2_O_2_ production indicating a probable 2x2e^−^ transfer mechanism. The performance of the air-breathing cathode was enhanced adding GNS or Fe-AAPyr separately and simultaneously. The addition of GNS and Fe-AAPyr concurrently led to the higher output of the investigation that was 235 ± 1 μW cm^−2^. The presence of only Fe-AAPyr had 8% lower power density (218 ± 5 μW cm^−2^). The addition of GNS within the cathode containing Fe-AAPyr increased the overall performance by 8%. AC was the baseline with a power density of 103–105 μW cm^−2^. The addition of the sole GNS led to the performance by 50% to a power density of 150 ± 5 μW cm^−2^.
